# Reductive amination of ω-conotoxin MVIIA: synthesis, determination of modification sites, and self-assembly

**DOI:** 10.1007/s00726-023-03366-2

**Published:** 2024-03-30

**Authors:** Xiufang Ding, Yue Wang, Sida Zhang, Ruihua Zhang, Dong Chen, Changcai Liu, Jianfu Xu, Long Chen

**Affiliations:** 1 State Key Laboratory of NBC Protection for Civilian, Beijing, 102205 China; 2 Beijing Key Laboratory of Bioprocess, College of Life Science and Technology, Beijing University of Chemical Technology, Beijing, 100029 China

**Keywords:** Reductive amination, MVIIA, Conotoxin, Modification, Self-assembly

## Abstract

Peptide drugs have disadvantages such as low stability, short half-life and side effects, which limit their widespread use in clinical practice. Therefore, peptide drugs can be modified to improve these disadvantages. Numerous studies have shown that alkyl-modified peptide drugs can self-assemble to prolong the duration of efficacy and/or reduce side effects. However, the commonly used solid-phase synthesis method for alkyl-modified peptides is time-consuming. To overcome this, a simple reductive amination reaction was employed, which can directly graft the alkyl chain to the peptide sequence and effectively avoid stepwise synthesis from C- to N-terminal with amino acids. In this study, ω-conotoxin MVIIA was used as the peptide drug, while myristic aldehyde was used as the alkylating agent. To obtain the maximum productivity of modified peptides, the molar ratio of peptide MVIIA to myristic aldehyde in the reductive amination reaction was optimized. Furthermore, the peptide modification sites in this reaction were confirmed by secondary mass spectrometry analysis. Besides, alkyl-modified peptide MVIIA was able to form micelles by self-assembly and improved stability in serum, which was related to our previous work where myristoylated peptide MVIIA micelles can improve the drug stability. Finally, this study was intended to provide a methodological basis for modifying the alkyl chain of peptide drugs.

## Introduction

Peptide drugs are gradually becoming the focus of new drug development, and they are highly valued by the pharmaceutical industry and hospitals, due to their many advantages such as high affinity, good selectivity, specific target, high safety (Fosgerau and Hoffmann 2015; Lee and Poh 2023). However, poor stability, short half-life and high plasma clearance restricted their usage as drug candidates (Lee and Poh 2023; Barman et al. 2023).

Conotoxins, also known as conopeptides, are a diverse group of peptides found in the venom of the marine cone snail that are used for prey capture and host defense (Craik and Adams 2007; Bjørn-Yoshimoto et al. 2020; Han et al. 2008). They have attracted interest in drug design due to their potent activity against a range of mammalian targets. ω-Conotoxin MVIIA is one of the best known conotoxins discovered to date (Patel et al. 2018). Meanwhile, MVIIA was approved by the U.S. Food and Drug Administration (FDA) in 2004 and is marketed under the name ziconotide (Prialt^®^) for the treatment of severe chronic pain (Patel et al. 2018; Garber 2005; Klotz 2006; Williams et al. 2008). It can inhibit the perception of painful stimuli by blocking N-type voltage-gated calcium (Ca_v_2.2) channels (Schmidtko et al. 2010; Snutch 2005; Gao et al. 2021). Despite the efficacy of MVIIA in the treatment of refractory pain, there are several limitations to its use as a therapeutic agent, including low bioavailability and stability (Newcomb et al. 2000), a narrow therapeutic window (Scott et al. 2002), and severe side effects (Penn and Paice 2000; Smith and Deer 2009). Therefore, the structure of MVIIA can be modified by external groups to improve its stability and/or reduce its side effects.

Alkyl chain modification of peptides has received much attention in recent years (Evans et al. 2020; Liu et al. 2019; Yang et al. 2023), since alkyl chain introduction renders peptides hydrophobic, which can induce peptide self-assembly and lead to the improvement of peptide drug stability (Gao et al. 2023; Ding et al. 2023; Yang et al. 2023). Our previous study showed that self-assembled micelles formed by N-terminal myristoylated MVIIA at high concentrations can prolong the duration of analgesic effect, improve serum stability, and significantly reduce or even eliminate side effects (Ding et al. 2023). However, the commonly used solid-phase synthesis method for alkyl-modified peptides is time-consuming. Therefore, in this study, a reductive amination reaction was attempted to couple myristic aldehyde to lysine/N-terminal cysteine of the ω-conotoxin MVIIA sequence. However, there are four lysines and one N-terminal cysteine in the ω-conotoxin MVIIA sequence, which should be confirmed and optimized in the reaction by tuning the molar ratio of peptide MVIIA to myristic aldehyde. Therefore, the modification sites of the C14-alkyl-modified peptides were diagnosed and the self-assembly properties were investigated.

## Materials and methods

### Materials and reagents

The peptide MVIIA used in this study (> 98% purity) was purchased from the Taijia Co., Ltd. (Hangzhou, China). All other chemical or biological reagents were bought from the Sigma-Aldrich Co. (St. Louis, MO, USA) unless otherwise stated.

### Reductive amination of MVIIA

#### Reaction optimization and preparation of single-site modified MVIIA

3 mg of ω-conotoxin MVIIA and 0.12 mg, 0.24 mg, 0.72 mg, and 2.41 mg of myristic aldehyde were weighed in molar ratios of 1:0.5, 1:1, 1:3, and 1:10, respectively, and dissolved in 1.5 mL of methanol containing 1% glacial acetic acid, and the mixed solution was stirred for 3 h at room temperature. Then, 0.28 mg NaBH_3_CN was added and the reaction was continued for 2 h with stirring. The reducing agent was quenched by adding 4–5 drops of saturated aqueous ammonium chloride solution and stirred magnetically for 30 min. After the reaction, 200 μL of the reaction solution was removed and the supernatant was centrifuged at 12,000 rpm for 20 min and analyzed by high-performance liquid chromatography (HPLC). The mobile phase used for HPLC was water containing 0.1% TFA in the aqueous phase and acetonitrile containing 0.1% TFA in the organic phase. The analytical column was a ZORBAX SB-C18 (4.6 × 250 mm, 5 μm). The detection wavelength was set at 220 nm, the flow rate was set at 1 mL/min, the column temperature was set at 25 ℃, and the injection volume was 20 μL. The gradient elution was performed by setting the organic phase ratio of 10–70%, and the liquid phase elution peak was collected; the collected eluate was analyzed by (mass spectrometry) MS with the following MS parameters: ion source of ESI, positive ion mode, and fragmentation voltage of 135 V. The synthesis was successful if the relative molecular mass was as expected. After the reaction, the separation was performed using preparative liquid phase with gradient elution at 35–80% organic phase ratio, and the eluate for the sample with the correct molecular mass corresponding to the time of elution peak (peak emergence time in the range of 25–45 min) was collected. Liquid chromatography was performed on a YMC-Pack ODS-A column (20 × 250 mm, 5 μm) at a flow rate of 6 mL/min, column temperature: 25 ℃, injection volume: 5 mL, detection wavelength: 220 nm, mobile phase A: H_2_O + 0.1% TFA, mobile phase B: ACN + 0.1% TFA. The collected eluate was removed from acetonitrile by rotary evaporation, and the remaining eluate was lyophilized under vacuum at – 80 ℃ to obtain peptide powder; the purity of the analyzed peptide samples was determined by the ratio of the elution peak with the correct molecular weight to the integrated peak area of all eluted peaks. The purity of the single-site modified MVIIA was found to be 95.64%.

#### Preparation of two-site modified MVIIA

For the preparation of the two-site modified peptide, ω-conotoxin MVIIA and myristoyl aldehyde were reacted in a 1:2 molar ratio, and gradient elution was performed by HPLC using an organic phase ratio of 10–80%. Other conditions were consistent with the single-site modified peptide synthesis method. The purity of the three two-site modified MVIIA 1, 2, and 3 was 92.64%, 91.32%, and 91.63%, respectively.

### Identification of modification sites for myristic aldehyde-modified peptides

Since there are multiple modifiable sites in the sequence of ω-conotoxin MVIIA, it is difficult to achieve site-specific modification, and further identification of the modification sites is required for the isolated modification products. ω-conotoxin MVIIA sequence contains three pairs of disulfide bonds that form a certain spatial structure. The bond energy of the disulfide bond is higher than that of the amide bond, which is difficult to fragment during collision-induced dissociation mass spectrometry, so the modified peptide should first be linearized before identification. The peptide disulfide bond is first opened by a reduction reaction and then alkylated to prevent the disulfide bond from reforming.

The modified peptide was dissolved at 0.8 mg/mL with 10 mM DTT and the reaction was carried out at 60 ℃ for 1 h. After the reaction, an equal volume of IAM solution was added and the reaction was continued at 25 ℃ for 1 h. Samples were desalted prior to mass spectrometry injection, i.e., the samples were eluted from the Monospin C18 desalting column with 80% acetonitrile-H_2_O solution (containing 0.1% TFA). The desalted samples were analyzed by liquid chromatography/quadruplex column with electrostatic field orbital trap mass spectrometry (Q Exactive focus MS). The liquid phase conditions were as follows: UV light absorption detector; Agilent Poroshell 120 SB-C18 column (4.6 × 100 mm, 2.7 µm); column temperature 45 ℃; flow rate 0.3 mL/min; injection volume 1 μL; mobile phases: ultrapure water solution containing 0.1% formic acid for the aqueous phase and acetonitrile solution containing 0.1% formic acid for the organic phase. The mass spectrometry conditions were: ion source was HESI (heated-ESI)-II; positive ion mode; scan range: 200–2000 *m*/*z* (resolution (R): 35,000); automatic gain control (AGC): MS1:3 × 10^6^, MS2:1 × 10^5^; NCE collision energy: 27 eV; Isolation window: 2.0 *m*/*z*; Max injection time: MS1:100 ms, MS2: 200 ms; Mass Spectrometry Fragmentation Mode: HCD mode.

### 1-Anilinonaphthalene-8-sulfonate (ANS) fluorescence binding assay

ANS was dissolved in DMF to make a starting solution of 20 mM. Peptides MVIIA, K2-MVIIA and K2-K4-MVIIA were prepared with 0.1 mM PBS (pH = 7.4) to form working solutions of 20 μM, 40 μM, 80 μM and 160 μM and incubated for 24 h at 37 °C. The final concentration of 20 μM ANS was added to various concentrations of peptide solutions. The excitation wavelength was set at 369 nm and the emission wavelength was set at 440–540 nm, and the fluorescence intensity was detected using a using a Hitachi F-4600 fluorescence spectrophotometer (Tokyo, Japan).

### Transmission electron microscopy (TEM) measurements

The peptides MVIIA, K2-MVIIA, and K2-K4-MVIIA were prepared in water as a 500 μM working solution and incubated for 24 h at 37 °C. 8 µL of the completed incubation working solutions of MVIIA, K2-MVIIA, and K2-K4-MVIIA was added dropwise to the individual carbon-coated copper grids and held for approximately 5 min before the samples were aspirated. Then, 8 µl of 2% aqueous sodium phosphotungstate solution (pH = 6.5) was dropped onto the copper grids and negative staining was performed on the MVIIA, K2-MVIIA, and K2-K4-MVIIA samples for 1 min, after which the samples were aspirated. Images were captured on a TEM at 100 kV using a JEOL JEM1200EX.

### Peptide stability in human sera

10% human serum was centrifuged at 14,000*g* for 15 min to separate the lipid layer from the serum, and the centrifuged supernatant was incubated at 37 °C for 15 min. Subsequently, peptides MVIIA, K2-MVIIA, and K2-K4-MVIIA were formulated into a 100 µM working solution in the supernatant and incubated at 37 °C. At 0, 1, 2, 4, 8, and 24 h, aliquots of peptides MVIIA, K2-MVIIA, and K2-K4-MVIIA were removed and mixed with an equal volume of 80% aqueous acetonitrile solution for 10 min at 4 °C. The mixture was centrifuged to remove the precipitate and the supernatant was analyzed by HPLC. The peak area of each sample was finally counted. The peak area of the 0 h sample was set to 100% peptide content and the percentage of peptide was calculated according to the formula: intact peptide (%) = peptide integral peak area at a time point/0 h peptide integral peak area × 100 (Zhou et al. 2017). Data were presented as the mean ± standard error (SEM) and were analyzed using one-way ANOVA with Dunnett’s T3 multiple comparison test.

## Results

### Reduction amination modification route of MVIIA

**Fig. 1:**
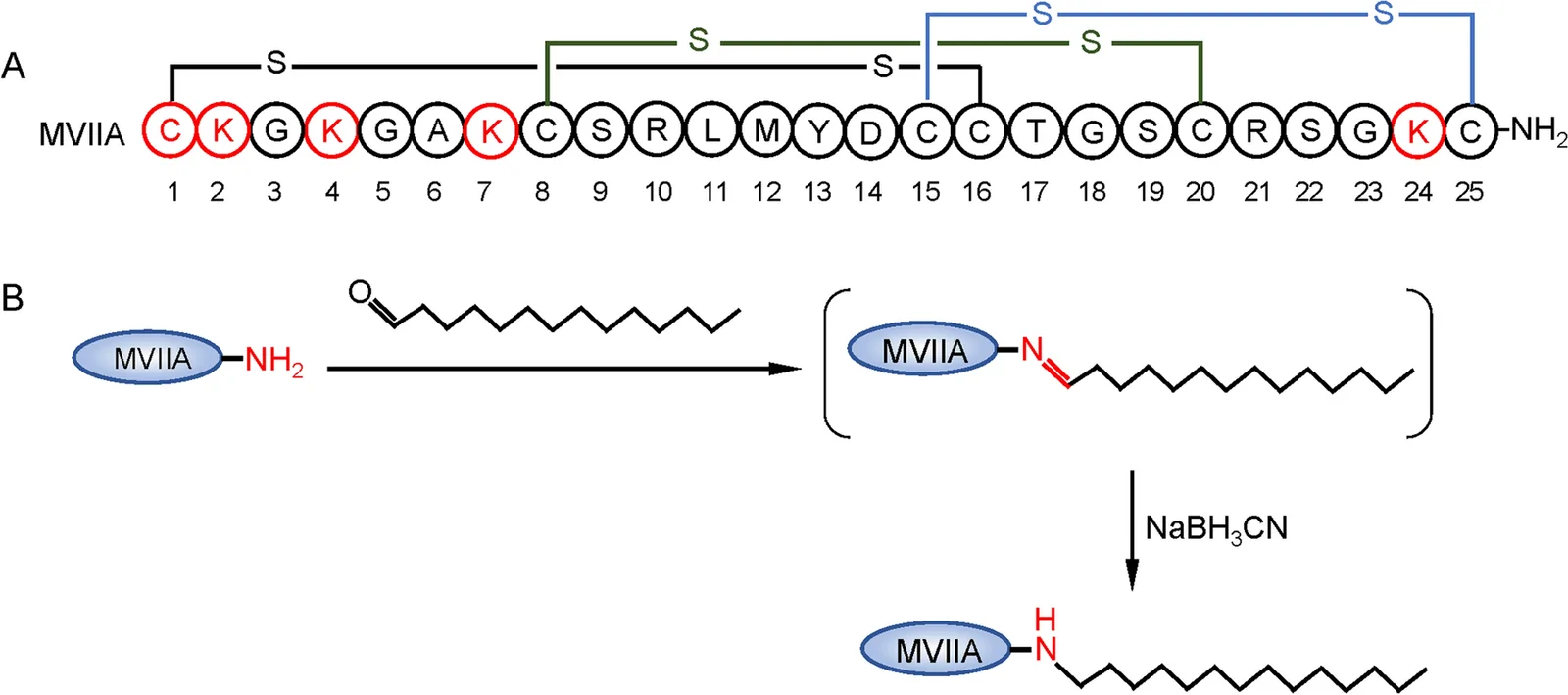
Structure and reductive amination route of MVIIA. (**A**) The structure of MVIIA with disulfide bonds. (**B**) Reductive amination of MVIIA

ω-Conotoxin MVIIA is a peptide of 25 residues with three pairs of disulfide bonds (Wermeling 2005). The structure of MVIIA is shown in Fig. 1A. There are four lysines and one N-terminal cysteine in the MVIIA sequence (Fig. 1A, marked in red), and their side-chain-free amino group can be used as a modification site. In addition, myristic aldehyde was chosen as a modifier because the aldehyde group is more reactive than the ketone with the amino group in the reductive amination reaction. The alkyl chain modification route of ω-conotoxin MVIIA in this study is shown in Fig. 1B. Myristic aldehyde reacted with the amino groups on the side chain of MVIIA to form Schiff bases, which were reduced by NaBH_3_CN to form covalent bonds to yield the alkyl chain-modified peptide.

During the reductive amination reaction, the molar ratio of peptide to myristic aldehyde, the modification reaction solvent, and the pH of the modification reaction are the main factors affecting the reaction. In this study, the modification reaction solvent was selected as methanol and the reaction pH was weakly acidic (pH = 5 or so), so the molar ratio of the reaction of ω-conotoxin MVIIA with myristoyl aldehyde was further optimized in this study.

### Optimization of the modification reaction

**Fig. 2:**
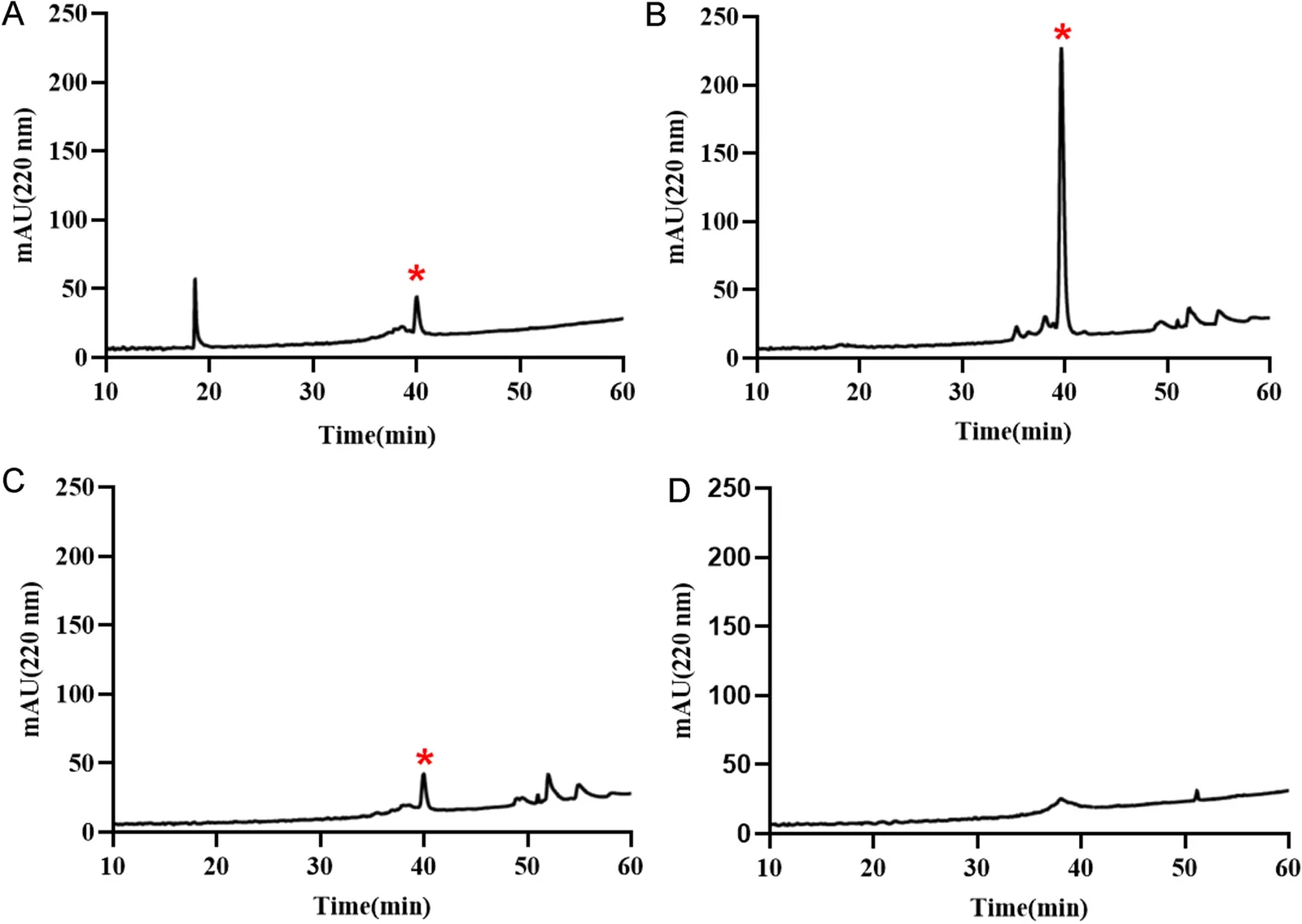
Liquid chromatograms optimized for reactions with molar ratios of (**A**) 1:0.5 (**B**) 1:1 (**C**) 1:3 (**D**) 1:10 for peptide and myristic aldehyde, respectively

After the reductive amination reactions of MVIIA and myristic aldehyde in the same environment at molar ratios of 1:0.5, 1:1, 1:3 and 1:10, the reaction products were detected by high-performance liquid chromatography. As shown in Fig. 2, the major elution peaks appeared at peak retention times of 20 min and 40 min. Based on the qualitative analysis of the peak retention time, the elution peak at 20 min corresponded to the peptide ω-conotoxin MVIIA before the reaction, while the eluate at the peak retention time of 40 min was considered to be the modified peptide. After the reaction between MVIIA and myristic aldehyde at a molar ratio of 1:0.5, the ω-conotoxin MVIIA peptide was not completely reacted and the intensity of the modified peptide was low (Fig. 2A). After the reaction of MVIIA and myristic aldehyde at a molar ratio of 1:1, the modified peptide peak was the main peak under the same conditions for 40 min, and a high signal peak intensity could be detected (200 mAu, Fig. 2B). However, when MVIIA and myristoyl aldehyde were reacted with excess myristic aldehyde (molar ratio of 1:3 and 1:10), the ω-conotoxin MVIIA peptide reacted completely, but the peak intensity of the modification product was detected at a low level for 40 min (Fig. 2C, D). It is speculated that this is due to over-introduction of myristic aldehyde, which makes the peptide more hydrophobic, forms solid aggregates and separates from the solvent. Therefore, a 1:1 molar ratio of MVIIA to myristic aldehyde for modification was a suitable choice to obtain the modified peptide.

### Modification site identification of single-site modified MVIIA

**Fig. 3:**
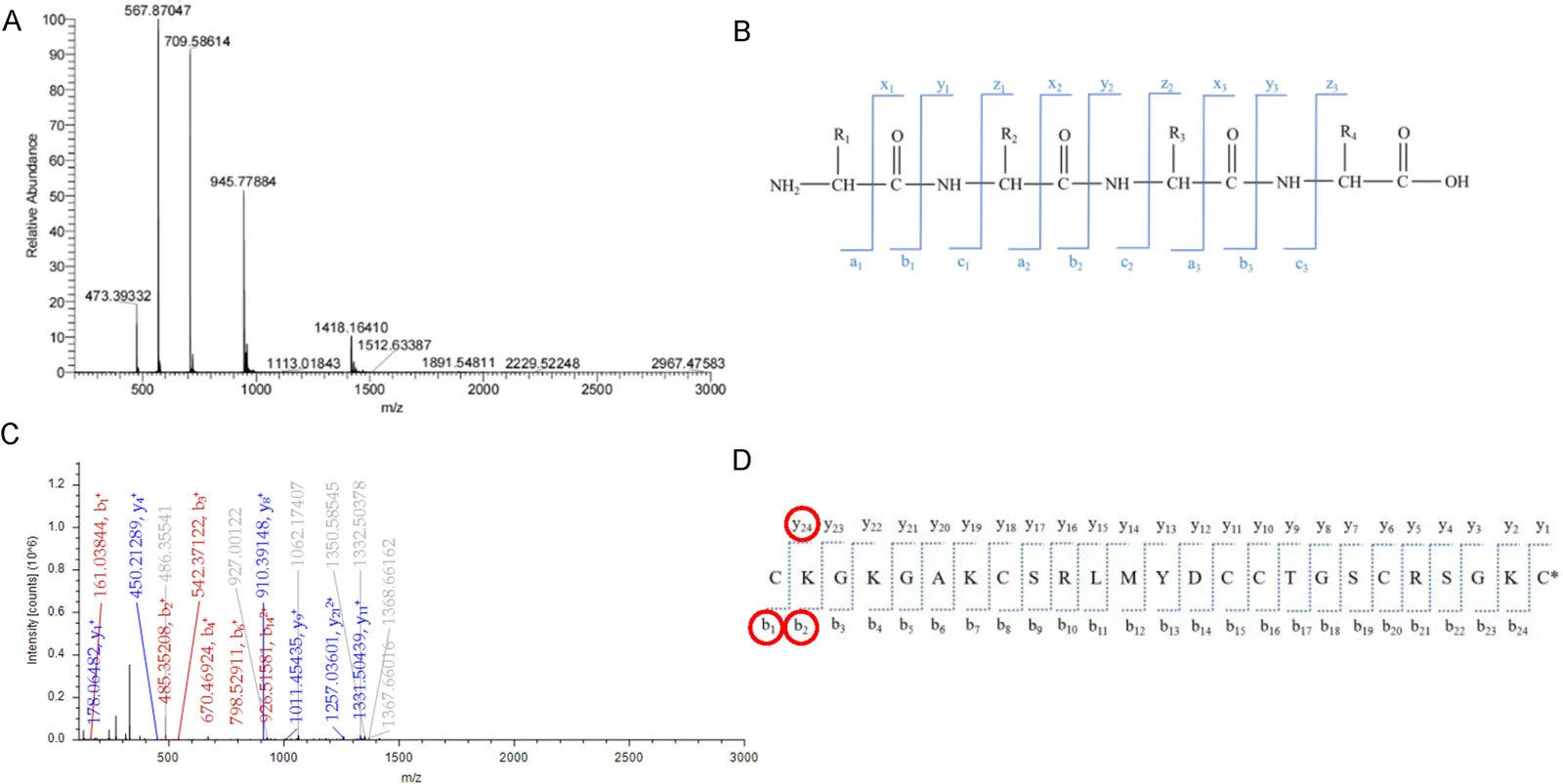
Modification site identification of single-modified peptides. (**A**) MS spectrum corresponding to the peak at 40 min (**B**) Schematic of theoretical peptide chain cleavage. When peptide chain was broken to generate ions, if the charge remained on the fragment at the N-terminus, we named a, b and c ions. If the charge remained on the fragment at the C-terminus, we named x, y and z ions. (**C**) MS/MS spectra of single-modified MVIIA. (**D**) Schematic of key fragment ions by program-assisted analysis

The expected theoretical relative molecular mass of the single-site myristic aldehyde-modified MVIIA was 2835.57 Da. The relative molecular mass of the reaction product at the peak retention time of 40 min was detected by mass spectrometry and was consistent with the expected relative molecular mass (Fig. 3A). Since the modification reaction was not site-specific, the modification site of the single-site myristic aldehyde-modified MVIIA peptide in Fig. 3A was further identified by secondary mass spectrometry. When the modified peptide passes through the primary mass spectrometer to produce differently charged parent ions, followed by selection of the target parent ions from the primary mass spectrometer-generated peptide in the secondary mass spectrometer, the peptide chain of the modified peptide is cleaved in the high-energy collision-induced dissociation cell. The theoretical peptide chain cleavage scheme is shown in Fig. 3B, where the a/x, b/y and c/z type fragment ions formed by the cleavage of the peptide bond between amino acids are indicated, and the b/y and a/x type fragment ions are more likely to be produced in the high-energy collision dissociation (HCD) fragmentation mode. Each peptide bond theoretically breaks to form b/y ions with similar peak intensities, but it is difficult to obtain the ideal trapezoidal series of ions in actual secondary mass spectra. If the secondary mass spectra contain key b/y series ions or some characteristic fragment ion peaks, the peptide sequence or modification site can be identified.

Since the MVIIA sequence contains three pairs of disulfide bonds, reduction to open the disulfide bonds is required for tandem mass spectrometry detection. In this study, the disulfide bond was first opened by reduction with DTT, and then alkylated with iodoacetamide (IAM) to avoid re-oxidation of the peptide to form a disulfide bond. The secondary mass spectra were further analyzed and the MS/MS mass spectral data obtained were processed using SEQUEST search software. The MS/MS spectrum of the 3-valent parent ion (*m*/*z* = 1061.50525) is shown in Fig. 3C. Schematic of key fragment ions by program-assisted analysis is shown in Fig. 3D. The software retrieved that the lysine at the second position (i.e., K2) of the peptide sequence is the modification site of the myristic aldehyde. Figure 3C shows that the fragments of b and y ions were abundant after cleavage, and the corresponding b ion peaks were b_1_^+^ (161.03844), b_2_^+^ (485.35208), b_3_^+^ (542.37122), b_4_^+^ (670. 46,924), b_6_^+^ (798.52911), and b_14_^2+^ (926.51581), and the major theoretical b ions were identified, and a number of abundant y series ion peaks, such as y_1_^+^, y_4_^+^, y_8_^+^, y_9_^+^, y_11_^+^, and y_21_^2+^, were also detected. When the peptide was unmodified, the possible monovalent b ions produced by the peptide MVIIA sequence had theoretical mass-to-charge ratios of b_1_^+^ (161.038473), b_2_^+^ (289.133436), b_3_^+^ (346.154899), b_4_^+^ (474.249862), b_5_^+^ (531.271326), b_6_^+^ (602.30844), etc. The b_1_^+^ (161.03844) in the MS/MS analysis was equal to the *m*/*z* value of the theoretical monovalent b_1_^+^ ion of MVIIA, indicating that the free amino group on the N-terminal cysteine was not modified. In addition, the difference between b_2_^+^, b_3_^+^, b_4_^+^, and b_6_^+^ in the b-series ions resolved by MS/MS and the theoretical b ion *m*/*z* in the unmodified case of MVIIA peptide is 196.22, which is equal to the isotopic molecular weight of -C_14_H_28_, and it is concluded that the tetradecyl chain at K2 is modified.

### Multi-site modification and identification of MVIIA

**Fig. 4:**
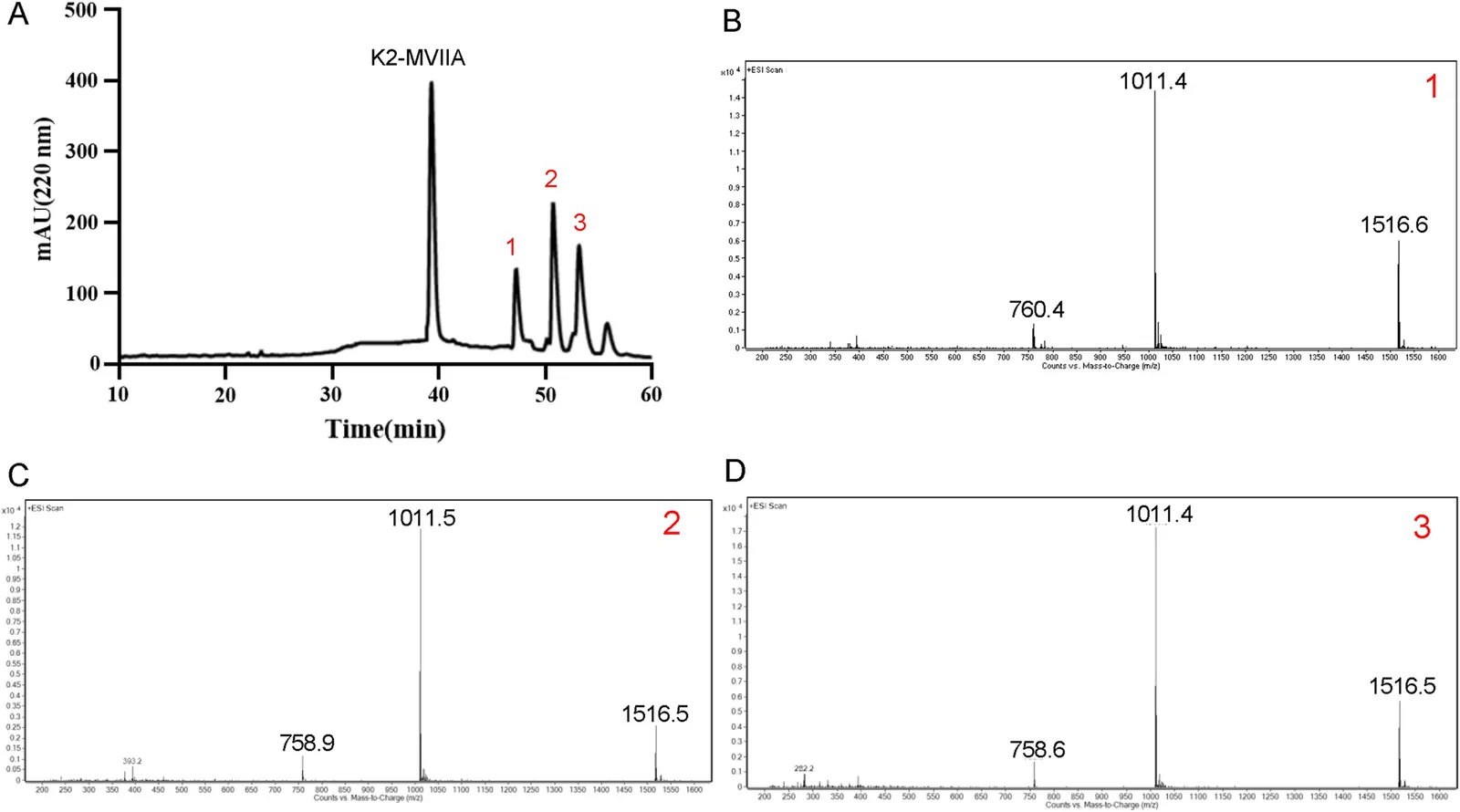
Isolation and characterization of the two-site modified peptide MVIIA. (**A**) The high-performance liquid chromatography (HPLC) of peptide MVIIA and myristic aldehyde with molar ratios of 1:2. (**B**) The MS spectrum of the two-site modified MVIIA 1. (**C**) The MS spectrum of the two-site modified MVIIA 2. (**D**) The MS spectrum of the two-site modified MVIIA 3

To further obtain multi-site modification of the alkyl chain-modified ω-conotoxin MVIIA, a modification reaction of MVIIA with myristic aldehyde at a molar ratio of 1:2 was performed, and the reaction products were detected by HPLC. As shown in Fig. 4A, four major elution peaks were collected for the reaction product with peak retention times of 40 min, 47 min, 51 min, and 53 min, respectively. The peptide corresponding to the 40 min peak retention time was identified as K2-MVIIA based on the peak retention time. The eluates collected at the 47 min, 51 min, and 53 min elution peaks were detected by ESI–MS. As shown in Fig. 4B, the observed molecular masses: [M+2H]/2: 1516.6, [M+3H]/3: 1011.4, were consistent with the expected molecular mass of 3031.94 for the two-site modified peptide. Similarly, the molecular masses observed in Fig. 4C ([M+2H]/2: 1516.5, [M+3H]/3: 1011.5) and Fig. 4D ([M+2H]/2: 1516.5, [M+3H]/3: 1011.4) were also consistent with the expected molecular mass of 3031.94 for the two-site modified peptide.

**Fig. 5:**
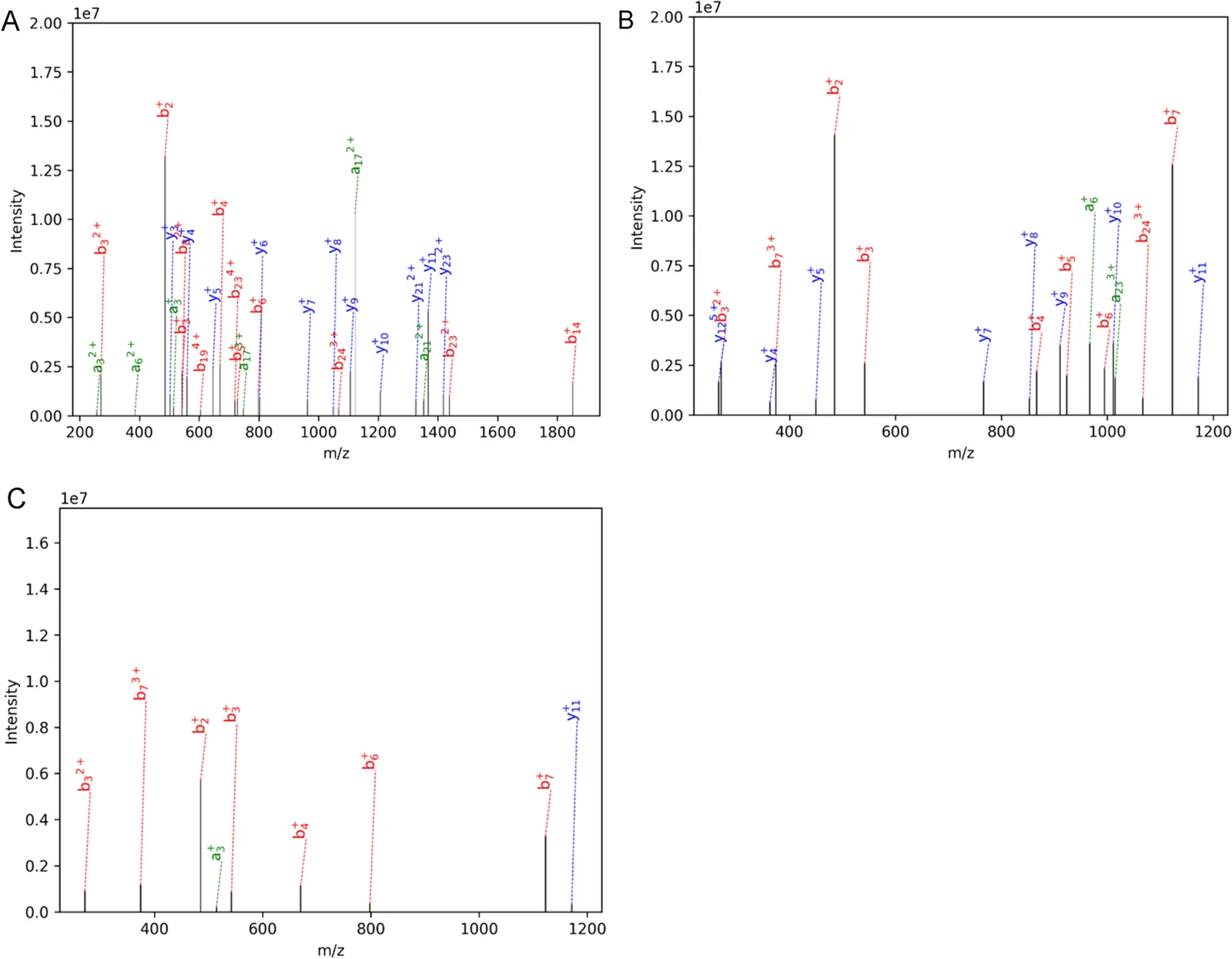
Identification by secondary mass spectrometry of the modification sites of the two-site modified peptide MVIIA 1 (**A**), MVIIA 2 (**B**), and MVIIA 3 (**C**)

After obtaining the two-site modified peptide, the modification sites were further identified. Similar to the identification of single-site modifications, the disulfide bonds were opened by reduction followed by alkylation and analyzed by tandem mass spectrometry. The possible double modification sites are 1C2K, 1C4K, 1C7K, 1C24K, 2K4K, 2K7K, 2K24K, 4K7K, 4K24K, and 7K24K, and the software-assisted resolution results are summarized in Tables 1, 2, 3. The intensity mass/charge spectra are shown in Fig. 5. As shown in Fig. 5A, the matched b ion peaks were b_2_^+^, b_3_^+^, b_4_^+^, b_5_^+^, b_6_^+^, b_8_^2+^, etc., and also a series of y series ion peaks such as y_3_^+^, y_4_^+^, y_5_^+^, y_6_^+^, y_7_^+^, y_8_^+^, and y_9_^+^, and a ion peaks such as a_3_^+^, a_3_^2+^, a_6_^2+^, and a_17_^2+^. As shown in Fig. 5B, the matched b ion peaks were b_2_^+^, b_3_^+^, b_4_^+^, b_5_^+^, b_6_^+^, b_7_^+^, b_7_^3+^, b_24_^3+^, etc., and also a series of y series ion peaks such as y_4_^+^, y_5_^+^, y_7_^+^, y_8_^+^, y_9_^+^, y_10_^+^, and y_11_^+^, and a ion peaks such as a_6_^+^ and a_23_^3+^. As shown in Fig. 5C, the matched b ion peaks were b_2_^+^, b_3_^+^, b_4_^+^, b_6_^+^, b_7_^+^, b_7_^3+^, etc., and also y series ion peaks such as y_11_^+^, and a ion peaks such as a_3_^+^. Since the single modification site was identified as K2, showing that K2 was the preferred site for the reaction, it was easy to conclude that one of the two modification sites of the peptide was K2. In addition, by combining the ion matching data in Tables 1, 2, and 3, the three two-site modified peptides were identified as K2-K24-MVIIA, K2-K4-MVIIA, and K2-K7-MVIIA, respectively.

**Table 1:** Analysis results of two-site modified MVIIA 1

Modification sites	Total number of matched ions	Number of matched characteristic ion species	Number of matching signature ions
2K24K	29	5	24
1C24K	30	4	19
4K24K	23	5	23
7K24K	18	3	9
1C7K	18	5	11
2K7K	17	6	16
1C4K	15	5	10
2K4K	14	6	12
4K7K	11	6	11
1C2K	10	2	1

**Table 2:** Analysis results of two-site modified MVIIA 2

Modification sites	Total number of matched ions	Number of matched characteristic ion species	Number of matching signature ions
2K4K	19	3	11
1C4K	20	3	8
1C7K	17	4	13
1C2K	17	0	0
2K7K	16	4	15
4K7K	13	4	13
1C24K	10	3	6
2K24K	9	3	8
4K24K	6	3	6
7K24K	5	3	5

**Table 3:** Analysis results of two-site modified MVIIA 3

Modification sites	Total number of matched ions	Number of matched characteristic ion species	Number of matching signature ions
2K7K	9	4	9
1C7K	10	4	6
1C4K	9	3	6
1C24K	9	2	3
2K4K	8	3	7
2K24K	8	2	6
4K7K	5	4	5
1C2K	5	0	0
4K24K	4	2	4
7K24K	3	3	3

### Self-assembly of single-site modified MVIIA and two-site modified MVIIA in aqueous solution

**Fig. 6:**
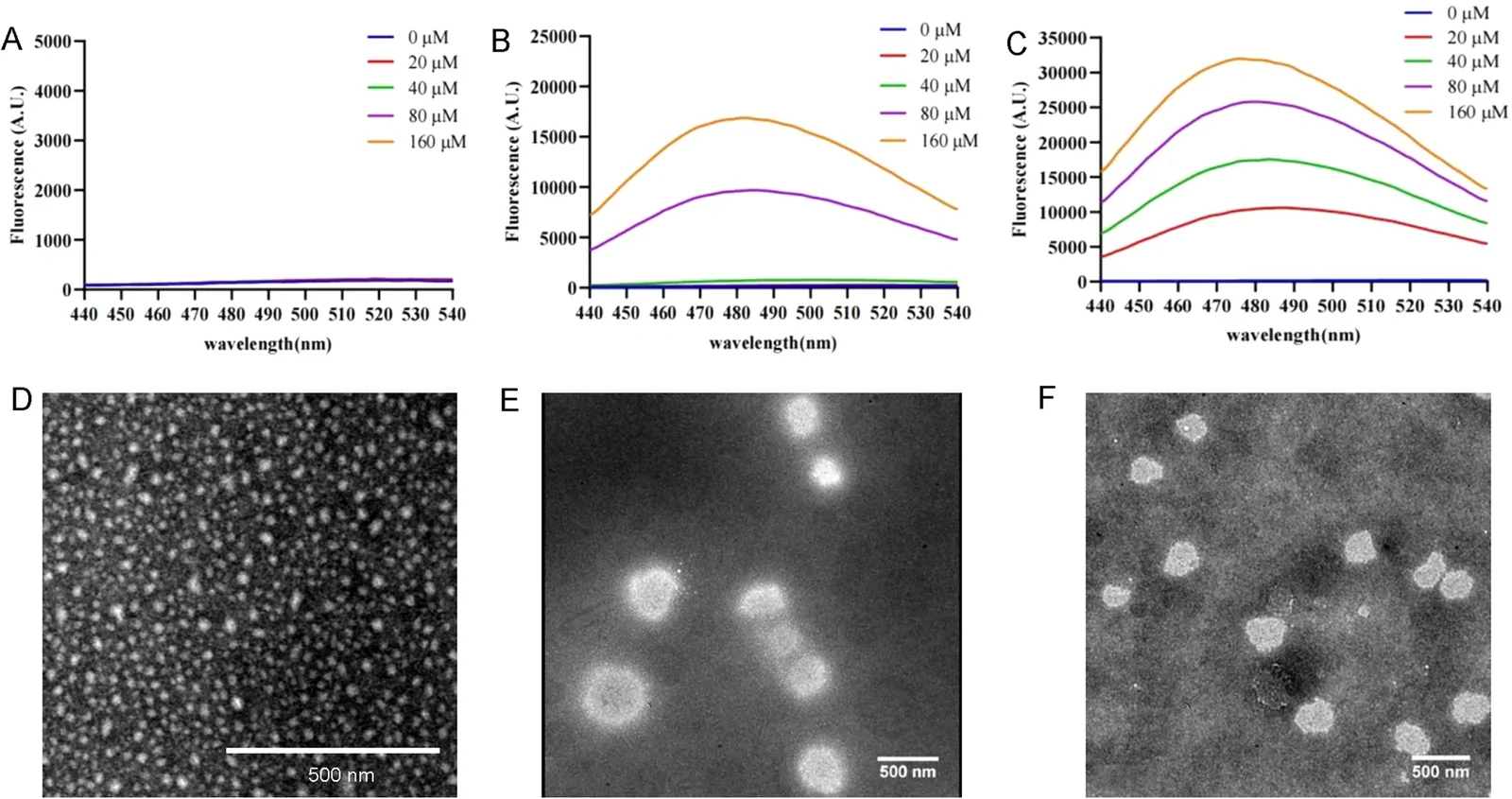
Characterization of aggregates formed by MVIIA, K2-MVIIA, K2-K4-MVIIA. The anionic 1-anilinonaphthalene-8-sulfonic acid (ANS) fluorescence traces for MVIIA (**A**), K2-MVIIA (**B**) and K2-K4-MVIIA (**C**). Transmission electron microscopy (TEM) images of MVIIA (**D**), K2-MVIIA (**E**), and K2-K4-MVIIA (**F**) aggregates at 37 °C for 24 h

The increase in hydrophobicity after the alkyl chain attached to MVIIA may promote the self-assembly of the peptide, so the self-assembly ability of the peptide was further verified in this study, in which K2-K4-MVIIA was selected as a representative of the two-site modified peptide. Peptides MVIIA, K2-MVIIA and K2-K4-MVIIA were incubated in the same self-assembly environment (physiological pH of 7.4 and temperature of 37 °C) for 24 h. Anionic 1-anilinonaphthalene-8-sulfonic acid (ANS) is a hydrophobic fluorescent dye with high affinity for the hydrophobic surface of peptides or proteins and can be used as a molecular probe to detect the self-assembly ability of peptides or proteins (Younan and Viles 2015; Lindgren et al. 2005). As shown in Fig. 6B, C there was a significant increase in fluorescence intensity with increasing concentrations of peptides K2-MVIIA and K2-K4-MVIIA. In addition, the highest peak of ANS emission shifts from 510 nm to near 480 nm, i.e., a blue shift of its own emission spectrum, indicating that ANS binds to the assembled K2-MVIIA and K2-K4-MVIIA. At the same concentration, the fluorescence intensity of K2-K4-MVIIA was higher than that of K2-MVIIA, while MVIIA showed almost no increase in fluorescence intensity (Fig. 6A), demonstrating that the two-site modified peptide K2-K4-MVIIA has a higher self-assembly capacity than the single-site modified peptide K2-MVIIA, while both modified peptides have a higher self-assembly capacity than MVIIA. The incubated peptide samples were negatively stained, and the self-assembled morphological structures of the peptides were observed by transmission electron microscopy (TEM, Fig. 6D, E, F). The experimental results showed that the peptides K2-MVIIA and K2-K4-MVIIA could form nanomicellar structures (Fig. 6E, F). Peptide MVIIA formed oligomers under the same conditions (Fig. 6D).

### Serum stability of single-site modified MVIIA and two-site modified MVIIA

**Fig. 7:**
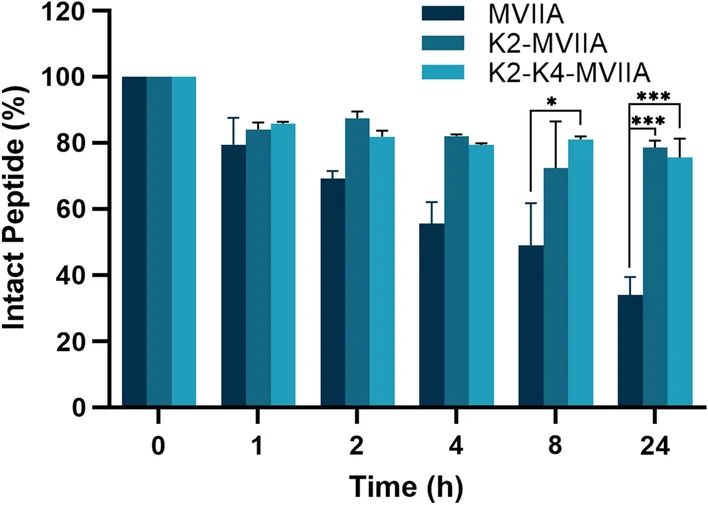
Degradation kinetics of peptides MVIIA, K2-MVIIA and K2-K4-MVIIA in human serum. Data are presented as mean ± SEM, *n* = 4. *p ≤ 0.05 and ***p ≤ 0.001 by one-way ANOVA with Dunnett’s T3 multiple comparison test

Natural peptides, despite their potential utility as therapeutic agents, have a short duration of activity in vivo due to low stability to protein hydrolysis, and one way to overcome these drawbacks is to use modified peptides (Gentilucci et al. 2010). As shown in Fig. 7, the half-life of peptide MVIIA in 10% human serum was about 8 h. However, the modified self-assembled peptides K2-MVIIA and K2-K4-MVIIA were more stable in 10% human serum, and the peptides K2-MVIIA and K2-K4-MVIIA remained intact for 78.68% and 75.61%, respectively, after 24 h (Fig. 7). This suggests that the micelles formed by self-assembly of modified peptides K2-MVIIA and K2-K4-MVIIA may protect the peptides from serum protease degradation.

## Discussions and conclusions

Peptide drugs have the advantages of high affinity, good specificity, and clear biological mechanisms and functions (Fosgerau and Hoffmann 2015; Lee and Poh 2023). However, their weaknesses such as poor stability, short half-life and in vivo instability severely limit their widespread use (Lee and Poh 2023; Barman et al. 2023). For example, the conotoxin drug MVIIA is clinically used for refractory chronic pain. However, MVIIA has low bioavailability and stability, a narrow therapeutic window, and severe side effects. How to solve these key problems is an important direction to be considered to promote the development of peptide drug industry. Modification of peptide drugs is widely used as an effective method and technique to solve the above problems.

Alkyl chain modification of peptides can increase the hydrophobicity of peptides and is a commonly used modification method for designing self-assembled peptides. Peptides containing one or more alkyl tails tend to form highly ordered nanostructures such as nanospheres, nanoribbons, twisted helices, nanotubes, and cylindrical nanostructures, and further lateral interactions also promote peptide hydrogelation (Rosa et al. 2023). In addition, it is shown that alkyl chain length and spatial exclusion between alkyl chains in the hydrophobic core limit the size and shape of nanospheres formed by peptide self-assembly (Muthusivarajan et al. 2020). Nanostructures formed by peptide self-assembly are believed to be effective in improving peptide stability, increasing resistance to enzymatic degradation, prolonging the half-life of peptide drugs, and reducing drug side effects (Ding et al. 2023; Zhou et al. 2017; Gao et al. 2023). Many approved fatty acid chain modification (similar to alkyl chain modification) drugs are in clinical use, and the fatty acids commonly used for drug modification are mainly myristic acid and palmitic acid. For example, Detemir is a new insulin analog prepared by removing Thr from the B30 position of human insulin and attaching a myristic acid side chain to lysine at the B29 position. After subcutaneous injection, the myristic acid side chain can effectively promote the formation of insulin hexamer and reversible binding with HSA, thus slowing down the diffusion of insulin in vivo and making the drug release slow (Kurtzhals 2007; Hordern 2006; Home and Kurtzhals 2006). Liraglutide, used in the treatment of type 2 diabetes and obesity, replaces the lysine at position 34 of human glucagon-like peptide 1 (GLP-1) with Arg and introduces a Glu-mediated 16-carbon palmitic acid side chain at position 26 Lys, which can form colloidal cluster-like aggregates at the subcutaneous injection site and is chemically stable and less susceptible to degradation (Ladenheim 2015; Nuffer and Trujillo 2015; Jacobsen et al. 2016; Iepsen et al. 2015).

Considering the presence of several lysines and an N-terminal cysteine in the MVIIA sequence, the free amino group of the lysine/cysteine side chain can be used as a modification site, and considering the better reactivity of the aldehyde group with the amino group during the reductive amination reaction, we chose the myristic aldehyde as a modifier. The reaction products with different molar ratios of peptide and myristic aldehyde were studied to optimize the reaction. Due to the non-site-specific modifications, the peptide modification sites were identified after the reaction using disulfide bond reduction and secondary mass spectrometry methods. Since previous studies showed that myristoylated MVIIA could self-assemble into micelles, which in turn prolonged the duration of peptide potency, increased stability, and reduced side effects of tremor and coordinated motor dysfunction in mice (Ding et al. 2023), this work also further investigated the self-assembly properties of the alkyl chain modification products obtained by the reductive amination reaction of peptides, and found that the single-site alkyl chain-modified peptide K2-MVIIA and the two-site alkyl chain-modified peptide K2-K4-MVIIA could self-assemble into micelles and improve serum stability. The shape of the two-site modified MVIIA micelles is more irregular than that of the single-site modified MVIIA, presumably due to the effect of the two alkyl chains. However, there was no significant difference in the improvement of serum stability between single-site and two-site modification. Comparison with previous work showed that both acylated and alkylated peptides self-assembled to form micelles, improving the serum stability of the peptides (Ding et al. 2023). This work provides a technical basis for the alkyl chain modification of peptide MVIIA, and the assembly of peptides into micelles will also provide a structural basis for its further study.

## Data Availability

The data presented in this study are available on request from the corresponding author.
